# A Cascade Attention Based Facial Expression Recognition Network by Fusing Multi-Scale Spatio-Temporal Features

**DOI:** 10.3390/s22041350

**Published:** 2022-02-10

**Authors:** Xiaoliang Zhu, Zili He, Liang Zhao, Zhicheng Dai, Qiaolai Yang

**Affiliations:** 1 National Engineering Laboratory for Educational Big Data, Central China Normal University, Wuhan 430079, China; zhuxl@ccnu.edu.cn; 2 National Engineering Research Center for E-Learning, Central China Normal University, Wuhan 430079, China; hzlzero@mails.ccnu.edu.cn (Z.H.); yql2020113547@mails.ccnu.edu.cn (Q.Y.)

**Keywords:** facial expression recognition, cascade attention, ResNeXt, pyramid feature, RAF-DB

## Abstract

The performance of a facial expression recognition network degrades obviously under situations of uneven illumination or partial occluded face as it is quite difficult to pinpoint the attention hotspots on the dynamically changing regions (e.g., eyes, nose, and mouth) as precisely as possible. To address the above issue, by a hybrid of the attention mechanism and pyramid feature, this paper proposes a cascade attention-based facial expression recognition network on the basis of a combination of (i) local spatial feature, (ii) multi-scale-stereoscopic spatial context feature (extracted from the 3-scale pyramid feature), and (iii) temporal feature. Experiments on the CK+, Oulu-CASIA, and RAF-DB datasets obtained recognition accuracy rates of 99.23%, 89.29%, and 86.80%, respectively. It demonstrates that the proposed method outperforms the state-of-the-art methods in both the experimental and natural environment.

## 1. Introduction

Human facial expression is one of the most natural and universal physiological signals by which humans can convey their feelings and behavioral trends. According to Ekman’s six basic cross-cultural emotions theory, facial expressions can be divided into six categories (i.e., anger, disgust, fear, happiness, sadness, and surprise) [1]. Most studies relevant to neutral emotions are based on the six basic emotions. Over the last 20 years, the field of computer vision has advanced rapidly, with facial expression recognition being a focal point due to its widespread application in human life such as human–computer interaction, virtual reality, intelligent course systems, and so on [2]. A variety of novel methods have greatly improved the accuracy of facial expression recognition. Among them, the mainstream methods of static facial expression recognition include traditional manual feature methods such as LBP [3] and SIFT [4]; nevertheless, the aforementioned traditional methods have difficulty extracting powerful temporal features hidden in facial images by manual descriptors. Because facial expression reflected in video sequences is a dynamic process, many studies now employ dynamic methods to learn face image features while incorporating face networks to extract temporal and spatial features of facial expression images [5]. Mengyi Liu et al. proposed a spatio-temporal model obtained from the dense low-level features of the video; subsequently, the generalized flow model is learned and fitted from all low-level features [6]. Hasani et al. created a network that extends the well-known 2D Inception-ResNet module, which is followed by a long short-term memory (LSTM) that classifies the sequences using these temporal relationships [7]. Nonetheless, the accuracy of facial expression recognition in video sequences is still influenced by lighting, deflection, occlusion, and other objective factors affecting image quality [8]. To address the issue, a variety of facial expression recognition methods [9,10,11] learn facial expression features by eliminating the interference caused by various interference factors such as posture, identity, and illumination, and have improved recognition performance for many public datasets collected in the laboratory or through various ways such as CK+ [12,13], MMI [14], Oulu-CASIA [15], SFEW/AFEW [16], FERPlus [17], AffectNet [18], EmotioNet [19], and RAF-DB [20,21].

Although the previously mentioned methods have some effects on expression recognition, there are some limitations; for example, eliminating interference factors may weaken some important facial features. As a result, researchers want to use the human visual mechanism (i.e., embed attention modules in neural networks to mimic human visual perception) to enable the neural network to ignore irrelevant information and focus more on the important information. For example, Jiyoung Lee et al. used recurrent attention in the spatial encoder and sequential decoder networks to improve the accuracy of facial expression recognition [22]. Jiaolong Yang et al. added double-layer attention blocks to the aggregation network, effectively improving the performance of the neural network for video facial recognition [23]. Qiangchang Wang et al. proposed a hierarchical pyramid diversified attention network that could enrich the feature context information and make the network more efficient in face recognition by considering hierarchical multi-scale local features and combining them with attention [24]. Even though the existing attention methods have contributed greatly to facial expression recognition, there is a problem of the insufficient utilization of spatial features, in addition, the method of attention to obtain the focal region also needs to be further improved. Consequently, there is still room for improvement in recognition accuracy (by extracting stereoscopic spatial information additionally) when they are used in a natural environment.

Fortunately, deep reinforcement learning (DRL) techniques have recently been proposed, which enables the artificial agents to learn both the knowledge and experience directly from the actual data. As demonstrated in [25], DRL, which integrates the concepts of reinforcement learning and deep learning, can lead to better application results in anomaly detection. In other words, DRL can augment spatial features in multi-layer convolutional networks, which illuminated the idea of this study initially.

Under such a background, in this study, a pyramid structure was added into the proposed network and the contextual information (provided by the multi-level structure of the pyramid) was used, thereby constructing context-aware features and strengthening the spatial features. Specifically, we proposed a cascade attention-based facial expression recognition network to solve the facial expression recognition problems in video sequences such as attitude, identity, head posture, lighting conditions, and occlusion. The cascade attention-based facial expression recognition network consists of three parts: (i) local and multi-scale-stereoscopic spatial context feature extraction module; (ii) cascaded attention module; and (iii) temporal sequential feature extraction module. Given a batch of the face image sequence, local spatial features will be extracted through the ResNeXt network [26] first. The high-level features of the ResNeXt network will be saved and input into the pyramid multi-scale-stereoscopic feature extractor during the extraction process. The two parts’ local and multi-scale-stereoscopic spatial context features are then superimposed and fused to form a complete spatial context. The entire spatial context is fed into the cascaded attention module to obtain the attention aggregation feature. Then, the attention aggregation features are input into the temporal sequential feature module to extract the temporal information. Finally, the basic seven (anger, contempt, disgust, fear, happiness, sadness, and surprise) facial expressions can be classified.

The main contributions of our work are summarized as follows:

(i) We used a two-branch network form to extract multi-scale-stereoscopic features of faces using the pyramid mechanism so that the network can focus on key regions of faces and thus improve the recognition accuracy.
(ii) We proposed a novel attention aggregation method for the feature-weighted aggregation of local and multi-scale-stereoscopic spatial context features to focus on regions that contribute more to facial expression recognition, and we investigated the efficiency of single attention and cascading attention blocks for feature aggregation.
(iii) We used cascading to combine the spatial feature extraction network and temporal feature extraction network to make the feature contextual information of facial expressions richer, which results in better recognition performance of the network.

The rest of this paper is organized as follows. Section 2 introduces the research of the attention aggregation method and the application of the pyramid feature. Section 3 describes the specific method of this research and provides the model’s overall framework. Section 4 describes the specific experimental process and the analysis of the results. The research is summarized in Section 5.

## 2. Related Work

### 2.1. Attention Mechanism

The attention mechanism has been widely used to enhance the performance of neural networks after SE-Net (the first channel attention mechanism) showed good performance [27]. Attention development can be roughly divided into two branches: (i) feature aggregation and (ii) a combination of channel attention and spatial attention. Y Li et al. proposed a CNN with the attention mechanism, which consists primarily of two parts (region segmentation and occlusion perception) to identify the occluded areas of the face and focus on the unobscured areas [28]; GE adopts deep convolution, explores spatial expansion, and implements feature aggregation [29]. In the second branch, Sanghyun Woo, Jongchan Park, and colleagues adopted channel attention and spatial attention modules in neural networks using the average pooling and maximum pooling methods and then sequentially combined these two attention mechanisms to improve feature aggregation [30]. ScSE calculated spatial attention using 2D convolution and then combined it with channel attention [31]. Wang Y. et al. proposed methods of time-series data (including text and video) classification using LSTM with multi-residual attention mechanism [32,33]. In A2-Net, a new method for image or video recognition based on NL block relation function was introduced [34]. Dual attention network for scene segmentation considers both NL-based channel attention and spatial attention for semantic segmentation [35].

### 2.2. Pyramid Feature

Pyramid is usually used as a multi-scale feature extractor [36]. The simplest pyramid feature is an image that goes through a convolutional layer for feature extraction, which is then fed into multiple pooling layers, each of which outputs a feature map so that different feature maps at multiple scales can be extracted.

In the ordinary feature extraction process, convolution operation and non-local attention operation are both used for feature extraction on the same scale of the image, which results in a common drawback without using the regional information relationship of different spatial on the image [37]. Because non-local attention units are placed on higher-level feature maps for feature extraction, long-term semantic information and correlation can be calculated [38]. Dongyoon Han et al. proposed a deep pyramidal residual network, which combines the idea of pyramid hierarchy with the residual network to effectively improve the ability of image classification [39], illuminating us to take full advantage of both attention mechanism and pyramid features.

## 3. Proposed Methodology

### 3.1. Method Overview

We proposed a multiple attention mechanism to classify the facial expression sequences in videos. In the preprocess period, we divided the video sequence into T parts and randomly selected one frame in each part, and then obtained the selected T-frame image sequence X as the input of the neural network to extract the facial expression features of faces.

(1a) $$X=\left\{x^{1},x^{2},\ldots ,x^{T}\right\},\left(T\leq N\right)$$

where

(1b) $$T=\left\{\begin{array}{c} v_{\mathrm{threhold}},\;\mathrm{if}\;N\geq v_{\mathrm{threhold}}=3\\ N,\;\mathrm{else} \end{array}\right.$$

In Equations (1a) and (1b), $x^{T}$ is a random selection of image frames in each part of the processed video sequence, and N denotes the total number of image frames after processing the video. It should be noted that the division of the T parts is determined by the size of N. In our study, we referred to the experimental results of the frame attention network and set $v_{\mathrm{threhold}}$ to 3 [40] (i.e., $v_{\mathrm{threhold}}=3$). When the total number of image frames obtained after processing the video is greater than or equal to 3 (i.e., N ≥ 3), the sequence of image frames is automatically divided into three consecutive parts, which will contain the starting or peak frames of the human facial expression, so three image frames are randomly selected in the three parts (one from each part) for further processing, and when the total number of image frames obtained after processing the video is less than 3 (i.e., N < 3), all the divided parts are processed (one from each part) (i.e., T=N). Our goal was to obtain a good dynamic characteristic representation and classification for the video image frame sequence $x^{T}$.

**Figure 1:**
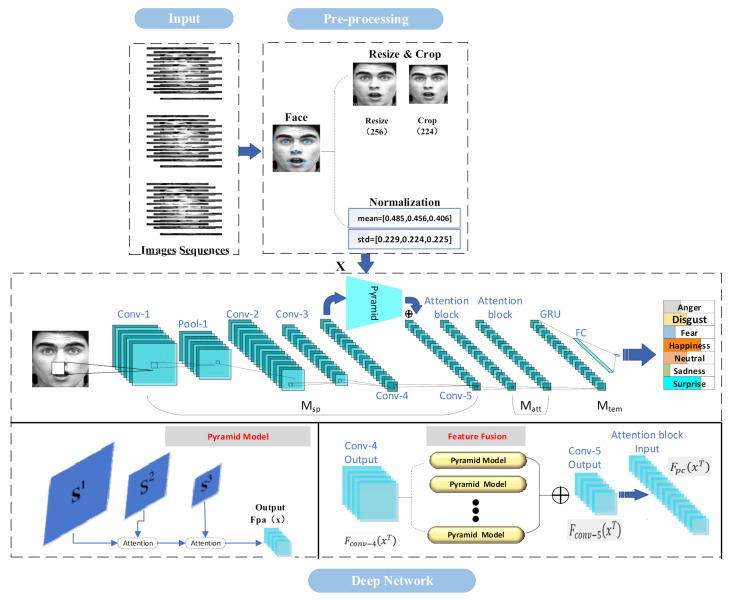
The architecture of the cascade attention-based facial expression recognition network model. We note that X represents the input to the network; Conv-1, Pool-1, Conv-2, Conv-3, Conv-4, and Conv-5 are the inner layers of the ResNeXt network; Pyramid denotes the pyramid feature extractor; $F_{pa}\left(x\right)$ denotes the output of the pyramid feature extractor; $F_{conv-4}\left(x^{T}\right)$ and $F_{conv-5}\left(x^{T}\right)$ represent the output characteristics of Conv-4 and Conv-5 of the ResNeXt network, respectively; $F_{pc}\left(x^{T}\right)$ denotes the input characteristics of the cascaded attention module; and ⨁ denotes the superimposed fusion operation of the features. The face image in this figure is from the CK+ database “S113”.

Our proposed network model, the cascaded attention-based facial expression recognition network, is based on a combination of multiple attentions and consists of three main modules: (i) a local and multi-scale-stereoscopic spatial context feature extraction module $M_{sp}$ to extract features in the spatial dimension (we note that three different scales were used in our study where the downsampling parameters were [1.0, 0.9, 0.8] to extract 3-scale pyramid features), which was similar to [41]; (ii) a cascading attention module $M_{att}$ to extract attention features; and (iii) a temporal sequential feature extraction module $M_{\mathrm{tem}}$ to extract features in the temporal dimension. The model structure is shown in Figure 1.

As shown in Figure 1, the local and multi-scale-stereoscopic spatial context feature extraction module $M_{sp}$ selects a variant of the residual network, the ResNeXt network, as the local and multi-scale-stereoscopic spatial context feature extractor and inputs the extracted local and multi-scale-stereoscopic spatial context features into the cascaded attention module $M_{att}$. $M_{att}$ uses a two-layer attention cascade to learn the attention weights from the local and multi-scale-stereoscopic spatial fusion features. The temporal sequential feature extraction module $M_{tem}$ takes the cascaded attention features as input and extracts the temporal features using the gate recurrent unit (GRU). Finally, the temporal features are fed to the fully connected layer to output the classification results.

### 3.2. Local and Multi-Scale-Stereoscopic Spatial Context Feature Extraction Module

As an advanced version of the traditional local spatial feature, multi-scale-stereoscopic spatial context is proposed in this study, which correlates the facial image regions that are concentrated across three different scales, and then used this correlation to extend the local spatial feature.

The local and multi-scale-stereoscopic spatial context feature extraction module uses the ResNeXt-50 network as the backbone network and incorporates pyramidal features. ResNeXt is a hybrid of ResNet [42] and Inception [43], and it learns the local spatial context features using grouped convolution. When a deep learning network reaches a certain depth, it encounters the vanishing gradient and exploding gradient problems, causing the network to lose its original performance. In addition, shortcut connections do not generate additional parameters to increase the learning pressure of the network and do not increase the computational complexity of the network, thus ensuring the performance of the network. The various convolutional branches in Inception networks that use grouped convolution have many hyperparameters that are especially difficult to adjust. The ResNeXt incorporates a simplified Inception concept based on the powerful residual network, which not only eliminates the effect of inception containing many hyperparameters, but also highlights the benefits of ResNet. As a result, the network can effectively prevent the deep network’s performance degradation. Meanwhile, the batch normalization [44] and dropout [45] layers included in the network effectively prevent the network overfitting and gradient problems. Table 1 shows the detailed structure of each layer of the ResNeXt network.

(2) $$F\left(x^{T}\right)=x^{T}+\sum_{i=1}^{C}J_{i}\left(x^{T}\right)$$

In Equation (2), $T_{i}$ denotes an arbitrary transform function; *C* is the size of the set of transforms to be aggregated in the network; and $F\left(x^{T}\right)$ denotes the high-level local spatial context features extracted by ResNeXt.

We input the T-frame image sequence X into the ResNeXt backbone network, saved the high-level features $F_{conv-4}\left(x^{T}\right)$ of Conv-4 of ResNeXt-50, and then extracted the multi-scale-stereoscopic spatial context features at different scales from its pyramidal feature extractor, which consists of two parts: the down-sampled pyramid and the attention layer. The saved high-level features of Conv-4 were downsampled, and the downsampling operation downsampled the high-level feature maps of Conv-4 to three different scale size feature maps $\left\{S^{1};\;S^{2};\;S^{3}\right\}$; then, the image information of multiple scales were subjected to the $F_{i}\left(\frac{H}{s_{i}}\times \frac{W}{s_{i}}\right)$ operation to obtain the feature maps and input to the scale attention for multi-scale feature extraction to obtain the spatial contextual features of feature maps of different scale sizes. The scale attention was used to weight spatial contextual features at different scales based on feature similarity at different scales; the spatial contextual features of each large-scale feature map was also fed into the scale attention, which was linked to adjacent small-scale feature maps, for feature weighting to obtain multi-scale-stereoscopic spatial context features $F_{pa}\left(F_{conv-4}\left(x^{T}\right)\right)$.

(3) $$W_{pa}\left(F_{conv-4}\left(x^{T}\right)\right)=\sum_{j=1}^{m}\sigma \left(f*g\left(F_{conv-4}\left(x^{T}\right),\;Concat\;\left(\theta \left(r,S^{i}\right)\right)\right)\right)$$

(4) $$F_{pa}\left(F_{conv-4}\left(x^{T}\right)\right)=\frac{1}{\sigma \left(S^{i}\right)}\theta \left(F_{conv-4}\left(x^{T}\right)\right)*W_{pa}\left(F_{conv-4}\left(x^{T}\right)\right)$$

where σ denotes the sigmoid function; $S^{i}$ denotes the i-th feature map of scaling; r represents the regional features of feature maps of different scale sizes; Concat means splicing operation; $F_{conv-4}\left(x^{T}\right)$ represents the facial expression texture feature; m represents the number of scaling scales; θ represents the spatial contextual feature transfer function of feature maps of different scale sizes; f∗g is the two-dimensional convolution operation; and $W_{pa}\left(F_{conv-4}\left(x^{T}\right)\right)$ represents the correlation weights of the context features in different regions of feature maps with different scale sizes.

### 3.3. Cascaded Attention Module

We now present the cascaded attention module to read the fused local and multi-scale-stereoscopic spatial context feature vectors from the ResNeXt network and the pyramid extractor and then generated a linear layer weight for them. Specifically, we let the local and multi-scale-stereoscopic spatial contextual feature vector $F_{pc}\left(x^{T}\right),$ which is fused by the local and multi-scale-stereoscopic spatial context feature extraction module, be input to an attention block, and then the attention block performs filtering of the features using a dot product operation with kernel k. The filtered feature vector is passed through the sigmoid function to generate the $A_{s1}$ global attention weights, which are then loaded onto the image. Because the important features have higher attentional weights than the secondary features, the loaded attentional face feature vector can globally highlight more important features of the face, resulting in the global key spatial domain contextual feature vector $FA_{s1}$. The upper three operations can be described as follows:

(5) $$A_{s1}\left(F_{pc}\left(x^{T}\right)\right)=\frac{1}{1+exp^{\left(K^{T_{*}Fpc}\left(x^{T}\right)\right)}}$$

(6) $$FA_{s1}\left(F_{pc}\left(x^{T}\right)\right)=\frac{1}{n}\sum_{t=1}^{n}A_{s1}\left(F_{pc}\left(x^{T}\right)\right)*F_{pc}\left(x^{T}\right)$$

where *n* is the number of initial input (facial expression images) of the network.

#### 3.3.1. Single Attention Block

**Figure 2:**
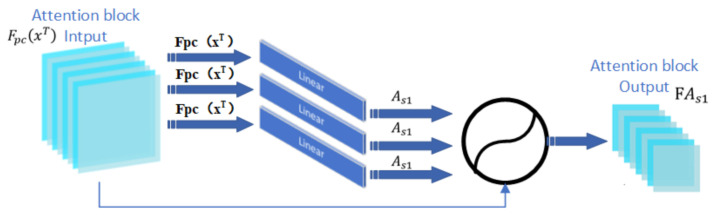
Attention block structure diagram. We note that $F_{pc}\left(x^{T}\right)$ is the output of the local and stereo space feature extraction module, $A_{s1}$ represents the attention weights of the facial expression images, and $FA_{s1}$ represents the weighted attention aggregated feature vector.

We first tried to add an attention block to the network to filter and weight the local and multi-scale-stereoscopic spatial context feature vectors. In this case, the vector K is a network-learnable parameter. The local and multi-scale-stereoscopic spatial contextual feature vectors $F_{pc}\left(x^{t}\right)$ of the three-frame face image sequence are input to the attention block, and the attention weights obtained after attention filtering are stitched into a set and loaded onto the face image feature vectors using the dot product method. The weighted attention features are aggregated using the attention superimposed fusion method to obtain the output feature $FA_{s1}$ of the final attention block. The single attention block composition is shown in Figure 2.

#### 3.3.2. Cascaded Attention Block

Cascaded attention blocks are feature aggregation after the extraction of multiple kinds of feature contexts using two-layer attention blocks. After extracting local and multi-scale-stereoscopic spatial context feature vectors as well as the global spatial context of faces, the second attention block performs feature aggregation using attention superimposed fusion after weighting the feature vectors. The process can be described using the following equation:

(7) $$A_{s2}\left(FA_{s1}\left(F_{pc}\left(x^{T}\right)\right)\right)=\frac{1}{1+exp\left(Q^{T_{*}FA_{s1}}\left(F_{pc}\left(x^{T}\right)\right)\right)}$$

(8) $$FA_{s2}\left(FA_{s1}\left(F_{pc}\left(x^{T}\right)\right)\right)=\frac{1}{n}\sum_{t=1}^{n}A_{s2}\left(FA_{s1}\left(F_{pc}\left(x^{T}\right)\right)\right)*FA_{s1}\left(F_{pc}\left(x^{T}\right)\right)$$

### 3.4. Temporal Sequential Feature Extraction Module

The GRU network [46] is used in the temporal feature extraction module. Compared with the LSTM [47], the GRU has one less unit of “gating”. The gradient problem in long-term memory and back-propagation is solved by the GRU, which has fewer parameters than the LSTM (long short-term memory). As a result, we employed a GRU network to read the aggregated feature vectors of cascaded attention blocks and extract the temporal features from the facial image sequences. The temporal sequential feature extraction module is a GRU network with 128 hidden neural units. The process can be described using the following equation:

(9) $$z_{t}=\sigma \left(W_{z}\cdot \left[h_{t-1},x_{t}\right]\right)$$

(10) $$r_{t}=\sigma \left(W_{t}\cdot \left[h_{t-1},x_{t}\right]\right)$$

(11) $${\tilde{h}}_{t}=tanh\left(W\cdot \left[r_{t}*h_{t-1},x_{t}\right]\right)$$

(12) $$h_{t}=\left(1-z_{t}\right)*h_{t-1}+z_{t}*{\tilde{h}}_{t}$$

In these equations, $x_{t}$ is the input to the GRU network; $z_{t}$ and $r_{t}$ are the outputs of the “update and reset gates,” respectively; ${\tilde{h}}_{t}$ is the new memory value; and $h_{t}$ represents the hidden state value.

## 4. Experiment and Results

### 4.1. Datasets

The CK+, Oulu-CASIA, and RAF-DB datasets were used in our experiments. The CK+ dataset is a collection of facial expressions gathered and compiled by a team of researchers from the University of Pittsburgh. The dataset contains 593 facial expression image sequences ranging from 10 to 60 frames in length, in which the facial expression gradually shifts from neutral to peak. There are 327 facial expression labels included in the 593 facial expression image sequences. The dataset for our study consisted of 327 face images with facial expression labels. The expressions in the dataset were classified into seven categories, namely, anger, contempt, disgust, fear, happiness, sadness, and surprise.

The Oulu–CASIA dataset is a publicly available facial expression dataset jointly published by Oulu University and the Chinese Academy of Sciences. The dataset contains face image sequences captured under three different lighting conditions: normal lighting, low lighting, and no lighting. The subjects were split into 50 Finns and 30 Chinese, with ages ranging from 23 to 58 years. Surprise, happiness, sadness, anger, fear, and disgust were the six categories for facial expressions.

The RAF–DB dataset is a large-scale database of facial expressions with 29,672 diverse facial images collected from the Internet. The dataset contains face image sequences captured under three different lighting conditions: normal lighting, low lighting, and no lighting. The subjects were split into 50 Finns and 30 Chinese, with ages ranging from 23 to 58 years. Surprise, happiness, sadness, anger, fear, and disgust were the six categories for facial expressions.

### 4.2. Data Preprocessing

In the face image data preprocessing period, facial expressions may be affected by factors such as head pose, lighting conditions, and occlusion (e.g., glasses, facial hair, or self-occlusion), which leads to the different performance of neural networks for different environmental facial expressions. Subsequently, an optimal preprocessing can effectively improve the recognition performance of facial expression [48].

In this study, we used the MTCNN method for the facial expression dataset, for all images using the standard MTCNN for the detection of the five landmark points (eyes, nose, and corners of mouth) of the face [49]. After performing similarity transformations, we obtained aligned facial expression images. Finally, the facial expression images were re-sized to 224 × 224 pixels and normalized. On the CK+ and Oulu–CASIA datasets, we ran a fivefold cross-validation test, dividing the original dataset equally into five sets of data, one of which was used as the validation set each time and the other four were used as the training set to train our network, and the final classification accuracy was the average accuracy obtained after five sets of tests. We present both the accuracy and average accuracy of the dataset on RAF–DB because the dataset has a training set and a test set, and there is an imbalance between the various categories of the RAF–DB dataset (i.e., the average accuracy is the average of the sum of all category accuracies).

### 4.3. Implementation Details

Our network model was based on the Pytorch deep learning framework and was experimented on an Ubuntu 16.04 system environment with an Intel i7-6800k CPU and an NVIDIA GTX1080Ti GPU. In the training phase of the network, we used a stochastic gradient descent optimizer and L2 regularization to avoid overfitting the network. The momentum of the network optimizer was set to 0.9, and the batch size was set to 8. The classification loss function used for the network weights was the cross-entropy loss function.

(13) $$Loss\;=\;\frac{1}{N}\sum_{i}L_{i}=-\frac{1}{N}\sum_{i}\sum_{c=1}^{M}y_{ic}log\left(p_{ic}\right)$$

We set the learning rate, different weight decay parameters, and different numbers of iteration rounds for different datasets. We set the learning rate to 0.001, the weight decay parameter to 0.0001, and the number of iterations to 100 for the CK+ dataset. We set the learning rate to 0.001, the weight decay parameter to 0.0001, and the number of iterations to 100 for the Oulu–CASIA dataset. For the RAF–DB dataset, we set the learning rate to 0.001, the weight decay parameter to 0.0005, and the iteration time to 200 epochs.

### 4.4. Gradient Class Activation Mapping Visualization

**Figure 3:**
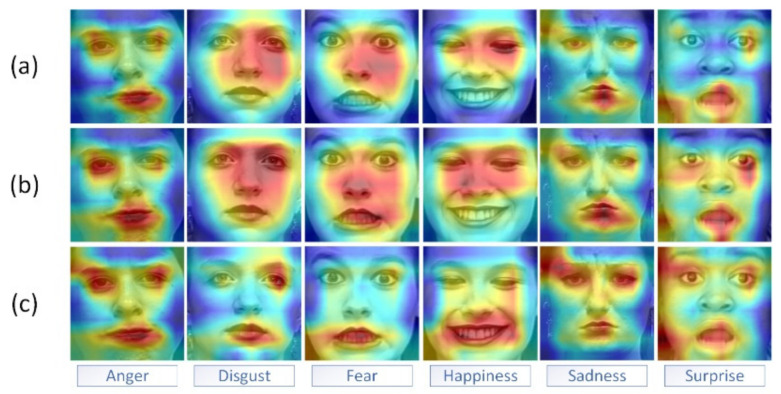
Visual comparison of gradient class activation mapping of CK+ (the three methods include the presence or absence of pyramids and single or cascading attention). From top to bottom, the visualization results are shown for the methods (**a**) without a pyramid but with cascading attention, (**b**) with a pyramid but with a single attention block, and (**c**) with a pyramid and cascading attention are shown in the expression images. We note that five subjects were included in these results: S055, S074, S106, and S111. The usage of their facial images is licensed. The use of their face images is licensed. Copyright reference: http://www.jeffcohn.net/Resources/ (accessed on 9 March 2021).

**Figure 4:**
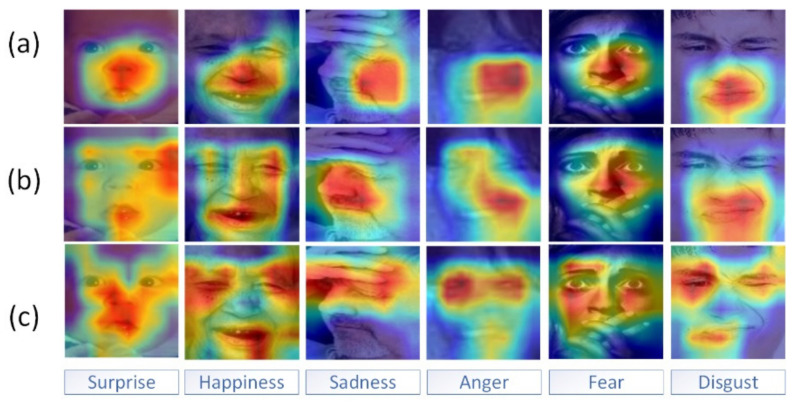
Visual comparison of gradient class activation mapping of RAF–DB (the three methods include the presence or absence of pyramids and single or cascading attention). From top to bottom, the visualization results are shown for the methods (**a**) without a pyramid but with cascading attention, (**b**) with a pyramid but with a single attention block, and (**c**) with a pyramid and cascading attention are shown in the expression images. The expression images from top to bottom show the visualization results for the methods without the pyramid but with cascading attention, with pyramid but with a single attention block, and with pyramid and cascading attention. Copyright reference: http://www.whdeng.cn/raf/model1.html (accessed on 8 June 2021).

In order to demonstrate the effect of having a pyramid extractor and the number of attention blocks on the network performance, we applied the xgradcam method [50]. As shown in Figure 3 and Figure 4, the red region represents the current region with a very high weight (i.e., the main area of attention of the neural network and the region that contributes the most to expression classification), and the blue-green region represents the current region with a lower weight.

As shown in Figure 3 and Figure 4, the first row shows the visualization of the gradient class activation mapping extracted from the last convolutional layer in the model without the pyramid module but with the cascaded attention; the second row shows the visualization of the gradient class activation mapping for the model with the pyramid module and the single attention block; and the third row shows the visualization of the gradient class activation mapping for the model with the pyramid and the cascaded attention module. In particular, when compared to the model without the pyramid feature extractor module, the network model with the pyramid feature extractor could focus more precisely on key regions of the face with variations such as the human mouth, nose, and eye regions. In comparison to the single attention block, the gradient category activation mapping visualization of the model with the cascaded attention block clearly showed that the red areas on the key areas of the face were darker, indicating that the cascaded attention block could highlight the key areas of the face better after feature aggregation, thus improving the recognition accuracy.

### 4.5. Experimental Results and Analysis

Table 2, Table 3 and Table 4 show the accuracy and average accuracy of the CK+, Oulu–CASIA, and RAF–DB datasets in the experiments, respectively.

A comparison of our proposed method with the state-of-the-art method [5,6,7,51,52,53] on the CK+ dataset is shown in Table 2. Our proposed method had an average accuracy of 99.23%. When compared to the two most accurate methods, GCNet and PHRNN-MSCNN, there was a 1.3% and 0.73% improvement, respectively. Table 3 shows a comparison of our proposed method and the existing state-of-the-art methods [5,51,52,53,54] on the Oulu–CASIA dataset. Our proposed method had an average accuracy of 89.29%, which was a 3.04% and 1.58% improvement over the two methods with the highest accuracy, PHRNN-MSCNN and FN2EN, respectively.

A comparison between our proposed method and the state-of-the-art methods [20,28,54,55,56] on the RAF–DB dataset is shown in Table 4. Facial expression images in the RAF–DB dataset are derived from the Internet and are influenced by age, gender, and race, head pose, lighting conditions, and occlusion (e.g., glasses, facial hair, or self-occlusion), making it a face dataset in a natural environment. Although classifying expressions on the RAF–DB dataset is difficult, our proposed method outperformed the state-of-the-art methods with 86.80% recognition accuracy and 78.37% average accuracy. These methods showed an improvement in terms of performance.

We further set up three control groups to explore the effect of the number of attention blocks and pyramid blocks on the performance of our proposed network. Here, control group 1 used ResNeXt-50 + cascaded attention block + GRU in the proposed multi-attention network; control group 2 used ResNeXt-50 + pyramid +single attention block + GRU in the proposed cascade attention based facial expression recognition network; and control group 3 used ResNeXt-50 + pyramid + cascaded attention block + GRU in the proposed cascaded attention-based facial expression recognition network. On the CK+ dataset (as shown in Table 2), control group 3 improved accuracy by 1.09% compared to control group 2, and control group 3 improved accuracy by 1.54% compared to control group 1. On the Oulu–CASIA dataset (as shown in Table 3), control group 3 improved its accuracy by 2.39% when compared to control group 2, and it improved its accuracy by 3.58% when compared to control group 1. On the RAF–DB dataset (e.g., Table 4), the accuracy of control group 3 improved by 0.78% when compared to control group 2, and the accuracy of control group 3 improved by 0.82% when compared to control group 1.

**Table 2:** Comparison on the CK+ dataset.

Methods	Accuracy
FN2EN [51]	96.80%
STM-ExpLet [6]	94.19%
LOMo [53]	95.10%
3D Inception-Resnet [7]	95.53%
GCNet [52]	97.93%
PHRNN-MSCNN [5]	98.50%
ResNeXt-50 + cascaded attention block + GRU	97.69%
ResNeXt-50 + pyramid + single attention block	98.14%
ResNeXt-50 + pyramid + cascaded attention block + GRU	99.23%

**Table 3:** Comparison on the Oulu–CASIA dataset.

Methods	Accuracy
LOMo [53]	82.10%
PPDN [54]	84.59%
GCNet [52]	86.11%
DCPN [57]	86.23%
PHRNN-MSCNN [5]	86.25%
FN2EN [51]	87.71%
ResNeXt-50 + cascaded attention block + GRU	85.71%
ResNeXt-50 + pyramid + single attention block	86.90%
ResNeXt-50 + pyramid + cascaded attention block + GRU	89.29%

**Table 4:** Comparison on the RAF–DB dataset.

Methods	Accuracy	Average Accuracy
FSN [58]	81.10%	72.46%
pACNN [55]	83.27%	Not provided
DLP-CNN [20]	84.13%	74.20%
ALT [56]	84.50%	76.50%
gACNN [28]	85.07%	Not provided
ResNeXt-50 + cascaded attention block + GRU	85.98%	77.66%
ResNeXt-50 + pyramid + single attention block	86.02%	77.84%
ResNeXt-50 + pyramid + cascaded attention block + GRU	86.80%	78.37%

The results of our experiments demonstrate the significant effect of cascaded attention blocks using feature fusion methods compared to single attention blocks in terms of aggregating multiple kinds of features and enriching feature contextual information. With the addition of a pyramid feature extractor to the network, the neural network can focus more on key parts of the face, thus improving the accuracy of facial expression recognition.

**Figure 5:**
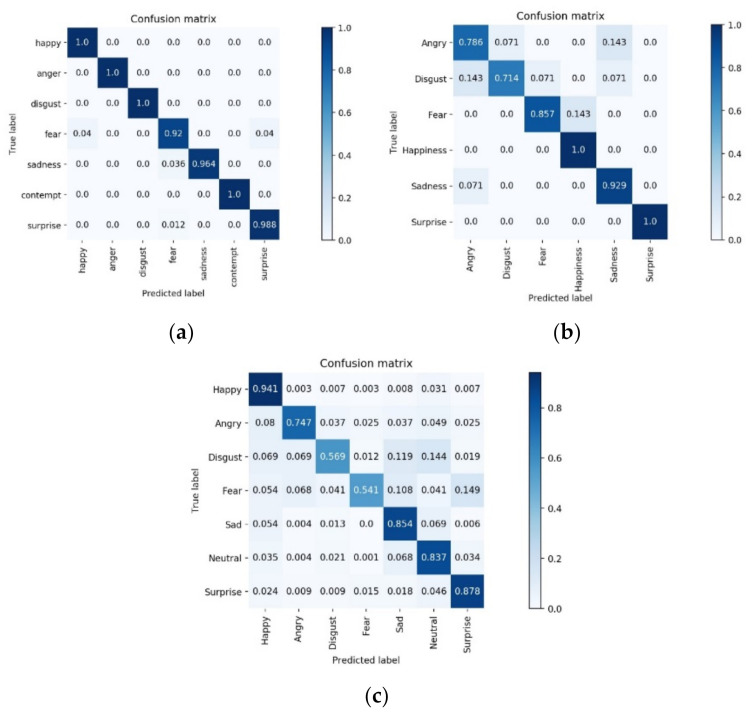
Confusion matrix of the network on the (**a**) CK+, (**b**) Oulu–CASIA, and (**c**) RAF–DB datasets.

We used the confusion matrix and ROC curves obtained from further model validation experiments to measure the performance of the model. The confusion matrix for network validation on different datasets after fivefold cross-validation is shown in Figure 5. The confusion matrix’s rows represent the true labels of the validation samples, while the confusion matrix’s columns represent the predicted labels of the validation samples. Furthermore, the accuracy on the diagonal line denotes the percentage of correct predictions for each category. As shown in Figure 5a, the prediction accuracy of each category on the CK+ dataset was high, but three categories, namely, fear, sadness, and surprise, were poorly predicted during the training process. On the Oulu–CASIA dataset (Figure 5b), happiness and surprise expressions had the highest recognition rates, while anger and disgust expressions had lower recognition rates. In particular, anger and disgust expressions were most likely to be confused in recognition. The recognition rate of happy expressions was higher on the RAF–DB dataset (Figure 5c) because the number of face images in the happy expression category was the largest in the RAF–DB dataset. The recognition rate of disgust and fear expressions was lower because the number of these two expressions was smaller and disgust expressions are easily confused with sadness and neutrality expressions, and fear is easily confused with sadness and surprise expressions.

The ROC curve is a general indicator of network classification performance. The horizontal coordinate of the curve represents the false positive rate, and the vertical coordinate represents the true positive rate. The ROC generally uses the area under the ROC curve (AUC) to analyze the model’s classification performance. AUC is the area enclosed by the ROC curve and the coordinate axis, and its value is typically between 0.5 and 1. The higher the AUC value, the better the model’s performance.

**Figure 6:**
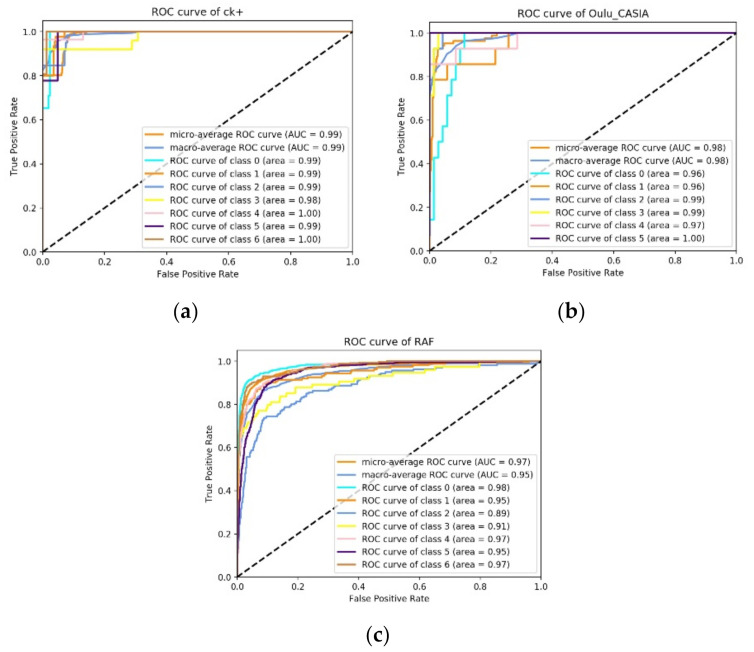
ROC curves on the CK+, Oulu–CASIA, and RAF–DB datasets. In each graph, the top two lines respectively represent the macro-average ROC curve and micro-average ROC curves of the dataset. In subplot (**a**) (i.e., the CK+ dataset), categories 0–6 correspond to happiness, anger, disgust, fear, sadness, contempt, and surprise, respectively; in subplot (**b**) (i.e., the Oulu–CASIA dataset), classes 0–6 correspond to expressions of happiness, anger, disgust, fear, sadness, neutrality, and surprise, respectively; in subplot (**c**) (i.e., the RAF–DB dataset), classes 0–6 correspond to happiness, anger, disgust, fear, sadness, neutrality, and surprise, respectively.

On the CK+ dataset (Figure 6a), the macro and micro seven-category average AUCs reached 0.99, indicating that this network had good performance on this dataset. On the Oulu–CASIA dataset (Figure 6b), the ROC curve and AUC both reached 0.98, indicating that the network performed well. The average AUC on the RAF–DB dataset (Figure 6c) exceeded 0.95, indicating that the model performed well with relatively high reliability in the natural condition.

## 5. Conclusions

To improve the performance of facial expression recognition under complex natural conditions, in this paper, a cascade attention-based network was proposed by combining the attention mechanism and pyramid feature. The main contribution of this study is that the proposed network not only makes full use of the contextual information to compensate for the underutilization of spatial features, but also further improves the performance of the attention mechanism and to a certain extent solves the problem of inaccurate localization of key regions of faces by neural networks. In particular, the pyramid feature was used in our study, which can (i) compensate for some high-level fineness characteristics by scaling operations and (ii) extract correlation information between scale-varying images, which aggregates features from different scales and thus can result in a richer feature set. As demonstrated by the visualized experimental analysis, by means of multi-scale-stereoscopic spatial context features, the proposed network can pinpoint the attention hotspots on particular regions with significantly dynamical changes (e.g., eyes, nose, and mouth) more precisely than the common attention strategy (i.e., the proposed network can track the areas that better represent facial expressions more precisely).

To be specific, the proposed network consists of the three following modules. The first module is a local and multi-scale-stereoscopic spatial context feature extraction module that extracts spatial context features using the ResNeXt-50 network and a pyramidal multi-scale-stereoscopic spatial context feature extractor. The cascaded attention module is the second module that performs weighted fusion of spatial features. The third module is the time series feature extraction module, which uses the GRU network to extract temporal features on the basis of fused features. Consequently, the multi-scale-stereoscopic spatial information of facial expressions is fused with the high-level spatial features of the residual network to enrich the spatial features to a great extent. The experimental verification on three publicly available datasets showed that the proposed model had good performance not only in the laboratory environment (i.e., with accuracy values of 99.23%, 89.29% on the CK+ and Oulu–CASIA datasets, respectively) but also in the complex natural environment (i.e., with an 86.80% accuracy on RAF–DB dataset).

To suit a more complex environment, our future work will include: (i) applying the augmentation technique to further improve the universality and robustness of the proposed network; (ii) optimizing the resource consumption of the network model in the stage of spatial feature extraction; and (iii) exploring more novel methods to further fuse spatial and temporal feature information.

## Figures and Tables

**Table 1:** Structure of ResNeXt-50.

Stage	Stage Setting	Output
Conv-1	7 × 7, 64, stride 2	(112, 112, 64)
Pool-1	3 × 3, MaxPool, stride 2	(56, 56, 64)
Conv-2	$\left[\begin{array}{c} 1\times 1,\;128\\ 3\times 3,\;128,\;C=32\\ 1\times 1,\;\;256 \end{array}\right]\times 3$	(56, 56, 128)
Conv-3	$\left[\begin{array}{c} 1\times 1,\;256\\ 3\times 3,\;256,\;C=32\\ 1\times 1,\;\;512 \end{array}\right]\times 4$	(28, 28, 256)
Conv-4	$\left[\begin{array}{c} 1\times 1,\;512\\ 3\times 3,\;512,\;C=32\\ 1\times 1,\;\;1024 \end{array}\right]\times 6$	(14, 14, 512)
Conv-5	$\left[\begin{array}{c} 1\times 1,\;1024\\ 3\times 3,\;1024,\;C=32\\ 1\times 1,\;2048 \end{array}\right]\times 3$	(7, 7, 2048)
Pool-2	Global Average Pooling	(1, 1, 2048)
Dropout	0.5	(1, 1, 2048)

## Data Availability

Data underlying the results presented in this paper are available in CK+ [12,13]. Data underlying the results presented in this paper are available in Oulu–CASIA [15]. Data underlying the results presented in this paper are available in RAF–DB [20,21].
